# Highly efficient and ultrahigh-resolution quantum dot light-emitting diodes via photoisomeric transformation

**DOI:** 10.1038/s41377-026-02246-0

**Published:** 2026-03-09

**Authors:** Chenglong Wu, Chengzhao Luo, Yonghuan Huo, Zixuan Chen, Chengze Xu, Xin Zhou, Zhiyong Zheng, Xinwen Wang, Zhenwei Ren, Yu Chen

**Affiliations:** 1 School of Optoelectronic Science and Engineering & Collaborative Innovation Center of Suzhou Nano Science and Technology, Soochow University, Suzhou, 215006 China; 2 National University of Singapore Suzhou Research Institute, Dushu Lake Science and Education Innovation District, Suzhou, 215123 China

**Keywords:** Lasers, LEDs and light sources, Photonic devices

## Abstract

The direct photopatterning technique provides a straightforward approach for high-resolution quantum dot (QD) patterns for next-generation displays. However, the extensively deteriorated QD optical properties deriving from the changes of the QD surface states and/or surroundings set substantial limitations in obtaining high-quality QD patterns and efficient electroluminescent devices. Here, we propose an ingenious and effective approach by utilizing the photoisomeric transformation from spiropyran to merocyanine for highly emissive QD patterns. We reveal the suppression of non-radiative energy transfer between QDs and the dissociative merocyanine for fast luminescence recovery. We achieve small-sized (0.8 μm), high-resolution (15,800 pixels per inch, PPI), high-fidelity (~100%), multicolor, and elaborated QD pixels, and showcase their good compatibility for CdSe/ZnS and perovskite QD pixel fabrication, as well as on both rigid and flexible substrates. These merits promote highly performing pixelated devices with a large luminance of 35,534 cd m^−^^2^ and a record efficiency of 24.5% at 6350 PPI among the direct photopatterning devices. Furthermore, we verify the wide applicability of the proposed strategy for high-performance pixelated perovskite devices with an efficiency of 13.8% at 1760 PPI. The above results confirm the great value of the proposed approach for high-quality QD patterns and high-performance pixelated devices.

## Abstract (web summary)

We reveal the suppression of non-radiative energy transfer between QDs and the photoisomeric merocyanine for greatly improved luminescence after patterning, achieving a record efficiency (24.5%) and resolution (15,800 PPI) among direct photopatterning approaches.

## Graphical abstract

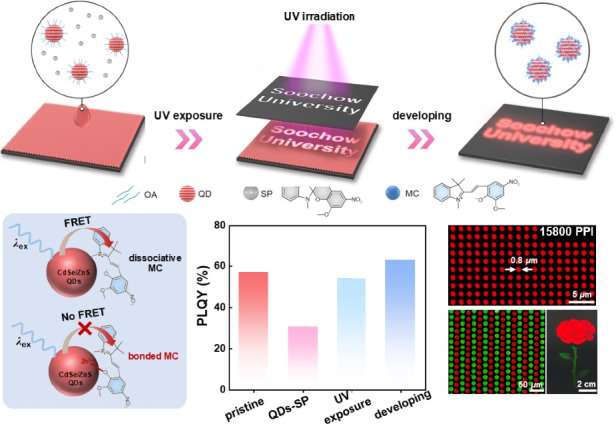

## Introduction

Colloidal quantum dots (QDs) have emerged as versatile luminescent materials due to their compelling properties of tunable emission wavelength, high color purity, good solution-processibility, and high photoluminescence quantum yields (PLQYs)^1–3^. For example, the applications of QDs in QDs-polymer luminescent composite, polychromatic emission, light fidelity communication, etc., have been significantly extended^4–6^. In particular, quantum dot light-emitting diodes (QLEDs) have received extensive interest, and significant breakthroughs have been achieved in device efficiency, brightness, and stability, enabling them to be ideal devices for potential applications in commercial displays^7,8^. Besides the progress in device performances, the patterned QLEDs are progressively desired to reach the requirements of ultrahigh pixel resolution (>2000 pixels per inch, PPI) for new-type displays, such as virtual reality displays, three-dimensional (3D) displays, and near-eye displays, in which high QD pixel density is required to convey the high flux information^9,10^. To pattern the QDs, a variety of techniques, including photolithography^11,12^, transfer printing^13–15^, inkjet printing^16,17^, dielectric and/or electrophoretic deposition^18,19^, etc., have been developed. However, some of these patterning methods suffer from inevitable drawbacks^20,21^, such as QD degradation, complex procedures, sophisticated apparatus, low fidelity, serious pixel crosstalk, and poor compatibility with the device preparation process, which undoubtedly restrict them in the fabrication of high-quality QD patterns, as well as the highly performing pixelated QLEDs^22–24^. Therefore, it is necessary to develop an effective and feasible approach to overcome the above hurdles and promote the implementation of the QD pixels and their patterned QLEDs toward practical application.

Recently, the direct photopatterning method has aroused considerable attention with a core concept of designing and/or modifying the QD ligand photochemistry to alter the solubility of QDs in developing agents for QD patterns^25^. Compared with conventional photolithography, the photoresist-free direct photopatterning technique avoids the resist-induced permanent QD damage and provides the opportunity to achieve high-luminescent QDs for the subsequent efficient device preparations. During direct photopatterning, the various ligand photochemistry properties are generally realized by the photochemical reactions, mainly involving the photo-crosslinking of QD ligands with functional linkers^26–29^ and the photo-induced detachment and/or decomposition of QD ligands by ligand exchange and radical reaction^30–32^. For example, the ligand crosslinkers with azide^33,34^ and carbene^35–37^ units have been developed to successfully interlock the ligands of adjacent QDs upon exposure to UV irradiation, yielding chemically robust and developing agent-insoluble QD patterns. Despite the microscale and uniform QD pixels achieved with the ligand crosslinking approach, the insulating nature of the polymeric ligands prohibits efficient charge transportation, thus hindering the further improvement of the device performance^38–40^. Fortunately, the detachment of the QD long-chain ligands provides an alternative approach to obtain the QD patterns with good electrical properties, solving the problem of deteriorated charge transportation in photo-crosslinked ligands. However, extensive studies reported deteriorated QD emission efficiency by removing the QD native ligands from the QD surface^41–43^. Even worse, there is a poor compatibility of the previous reports with the burgeoning metal halide perovskite QDs.

In this work, we conceive a novel and effective direct photopatterning approach by taking advantage of the photoisomeric transformation from spiropyran (SP) to merocyanine (MC) for high-quality QD patterns and demonstrate high emission efficiency for the developed QD patterns by suppressing the non-radiative energy transfer between QD and the dissociative MC. In combination with the passivation of QD surface defects, higher PLQYs than pristine QDs are observed for the developed QD patterns. The improvements in QD pattern PLQYs are much greater than those of previous analogs obtained with the direct photopatterning techniques. We exhibit the proposed approach in achieving small-sized (0.8 μm), high-resolution (15,800 PPI), high-fidelity (~100%), multicolor (RGB), and elaborated QD pixels, and demonstrate its good compatibility for CdSe/ZnS and perovskite QD pixel fabrications on both rigid and flexible substrates. These merits contribute to ultrahigh-resolution and highly efficient QLEDs with a remarkable luminance of 35,534 cd m^−^^2^ and a record external quantum efficiency (EQE) of 24.5% at 6350 PPI among the direct photopatterning devices. Moreover, we exhibit the broad applicability of the strategy for efficient pixelated perovskite QD devices (an EQE of 13.8% at 1760 PPI), validating its great value in the preparation of high-quality QD patterns and highly performing pixelated QD devices for practical applications.

## Results

### Principle of the light-driven QD patterning

**Fig. 1:**
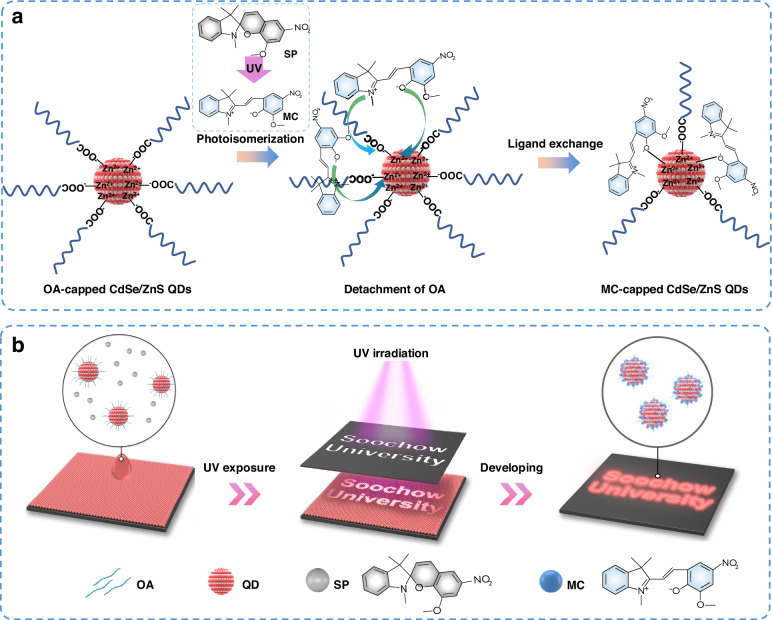
Illustration of the light-driven QD patterning process. **a** Schematic illustration of the photoisomeric transformation-induced ligand exchange between OA and MC. **b** Illustration of the QD patterning process with the successive steps of QD film preparation, UV exposure, and developing processes

The schematic diagram of the photoisomeric transformation-induced ligand exchange is shown in Fig. 1a. Generally, the CdSe/ZnS core-shell QDs are capped with massive organic ligands (e.g., oleic acid, OA), which enables the QDs to have good solubility in nonpolar and/or low-polar solvents, such as octane, hexane, toluene, etc. The SP molecule was conceived as a photosensitized agent due to the characteristic of UV light-driven structural transformation to the functional open-ring MC molecule^44,45^. The MC structure is displayed in Fig. 1a, which consists of functional sites of C–N^+^ and C–O^−^ deriving from the broken C–O bond of the SP molecule upon UV irradiation. As reported, the C–N^+^ cation is a kind of Lewis acid, which has a strong Coulomb force interaction with the nucleophilic carboxylate group (−COO^−^) of OA ligand^46^. The strong interaction promotes a loose and/or even broken bonding interaction between OA and the Zn^2+^ cation on CdSe/ZnS QD surface, thus resulting in a facile detachment of OA from the QD surface. Besides, we also reveal a stronger binding affinity of C–O^−^ to the Zn^2+^ than that of the −COO^−^ group in OA, which favors the direct ligand exchange from OA to MC. Both the strong interaction between C–N^+^ and OA and the high binding affinity of C–O^−^ to the Zn^2+^ prompt the detachment of the long-chain and oil-soluble OA ligands from the QD surface (illustrated in Fig. 1a). In detail, ~20% of the OA ligands were removed from the QD surface, resulting in a reduced OA ligand density from 4.0 to 3.2 nm^−^^2^ (Fig. S1, Table S1). While the bound MC ligands on the QD surface could well passivate the bare surface generated by OA removal. Encouragingly, the MC-capped/bonded QDs change the solubility of QDs from an OA-capped oil-soluble nature into an insoluble status, thus paving the way for QD patterning. As illustrated in Fig. 1b, the direct photopatterning of QDs is generally performed according to the following processes of (i) QD film deposition by spin-coating the mixed QD and SP solution, (ii) selective exposure of the QD film to UV light by utilizing a designed photomask, and (iii) elimination of the unexposed QDs with developing solvents to highlight the QD patterns. By employing different photomasks, we can obtain diverse QD patterns with various shapes and resolutions. More importantly, multicolor QD patterns can also be prepared by our strategy through the successive exposure and developing processes, as shown in the following part.

**Fig. 2:**
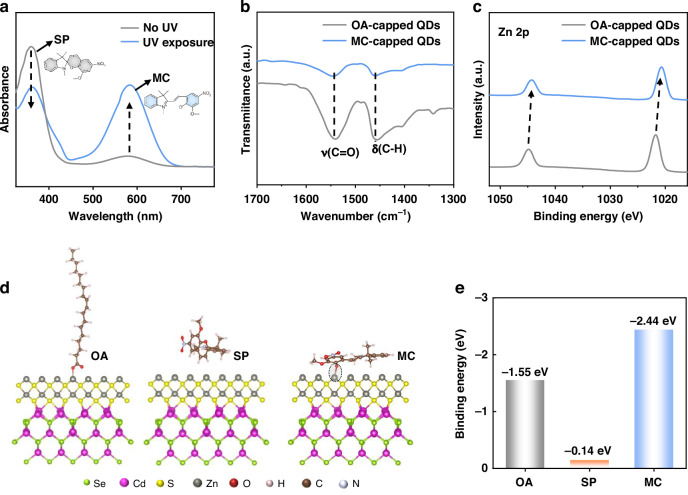
Characterization of the interaction between QDs and MC. **a** UV–vis absorption spectra of the SP solution before and after UV irradiation. **b** FT-IR and **c** XPS spectra of OA-capped pristine QDs and MC-capped QDs (i.e., SP-treated QDs after UV irradiation for 2 min). **d** Density functional theory (DFT) simulations of the binding energy of oleic acid, SP, and MC with uncoordinated zinc ions on the QD surface, and **e** the extracted binding energy values

To support the proposed principle for QD patterning above, abundant and sufficient experiments have been performed. The UV–vis absorption spectra of SP before and after exposure to UV irradiation are shown in Fig. 2a, where a characteristic absorption peak centered at 360 nm is observed for the SP molecule^44^, indicating a large optical bandgap of ~3.44 eV. Meanwhile, a distinct absorption peak at 583 nm assigned to the signal of the MC molecule is also found in pristine SP samples, suggesting the co-existence of MC in the SP molecules, which may be triggered by the ultraviolet component from the natural light. However, the absorption peak intensity of MC dramatically increases upon exposing the SP molecules to the UV light (365 nm), accompanied by a decreased peak intensity for the SP signal, showcasing the transformation of SP to MC under UV irradiation. To demonstrate the detachment of the oleic acid ligand from the QD surface, the measurements of Fourier transform infrared spectroscopy (FT-IR) were performed. As shown in Fig. 2b, the characteristic peak at 1540 cm^−1^ for pristine QDs is ascribed to the stretching vibration peaks of C=O from the carboxylate group in OA ligand. While the peak located at 1456 cm^−1^ is assigned to the scissoring stretching vibration of C–H from the long carbon chain of the OA ligand. Both these peak intensities largely decrease for the MC-capped QDs (i.e., SP-treated QDs under UV irradiation for 2 min, QDs-MC), indicating the great reduction of OA ligand after MC capping. The detachment of OA is further revealed by the deteriorated solubility of QDs (Fig. S2), where the well-dispersed OA-capped QDs in octane are observed to precipitate at the bottom of the solution after MC capping.

Consistently, the X-ray photoelectron spectroscopy (XPS) measurements were also performed to reveal the replacement of OA with MC (Fig. 2c), where the characteristic peaks at 1044.8 and 1021.7 eV for Zn 2p signal significantly shift toward lower binding energies of 1044.2 and 1020.7 eV after MC capping. The declined binding energy indicates the lowered oxidation state of Zn^2+^ due to the excessive electron donation from the C–O^−^ of MC. Besides the experimental results, we further theoretically investigate the mechanism of the ligand exchange from OA to MC by calculating their binding energies to the Zn^2+^ on the QD surface based on density functional theory (DFT) (Fig. 2d). The calculated binding energy between MC and Zn^2+^ is –2.44 eV, which is more negative than that of the OA with Zn^2+^ (–1.55 eV), indicating a stronger binding affinity of MC to the QD surface than that of OA. In other words, the capping of MC on the QD surface is more thermodynamically favorable than OA, thus driving the ligand exchange from OA to MC. Meanwhile, the binding energy between SP and the QDs was also calculated, where a small binding energy of merely −0.14 eV is obtained, indicating a significant difficulty for the binding of SP on the QD surface. Consequently, we have experimentally and theoretically demonstrated the detachment of oleic acid ligands from the QD surface by MC capping, which would contribute to high-quality QD patterns shown in the following part.

### Mechanism of the high emission efficiency for developed QD patterns

As extensively reported, the detachment of QD native ligands by ligand exchange alters the QD surface states and surroundings, inevitably resulting in poor luminescent performance^41,47^. Although substantial works have reported the passivation of QD defects for improved emission efficiency, very few studies show the full restoration of the QD optical properties due to other non-radiative recombination losses, such as the energy transfer between QDs and the capped photosensitive ligands. We demonstrate even higher emission efficiency for the developed QD patterns than pristine QDs through the effective suppression of the energy transfer-induced fluorescence quenching, as well as the MC-assisted defect passivation of the QDs in the following parts.

**Fig. 3:**
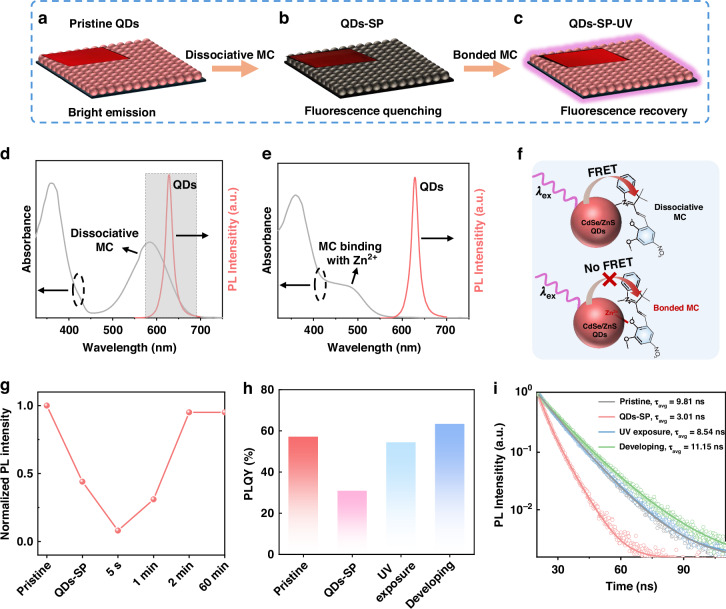
The QD optical properties during patterning process. **a**–**c** Schematic illustration of the fluorescence recovery process for the QDs. Presentations of d the overlapped spectra between the UV–vis absorption spectrum of dissociative MC molecules (UV irradiation: 2 s) and the PL spectrum of CdSe/ZnS QDs, and **e** the non-overlapped spectra between bonded MC molecules (UV irradiation: 2 min) and CdSe/ZnS QDs. **f** Illustration of the energy transfer process between QDs and unbonded (top)/ bonded (bottom) MC molecules. **g** The time-dependent PL intensity variations of the SP-treated QD films under UV irradiation. **h** The PLQY variations and **i** time-resolved PL spectra during the QD patterning process

The fluorescence recovery process of the QDs is illustrated in Fig. 3a–c and Fig. S3, where the OA-capped pristine QD films exhibit a bright red emission (Fig. 3a, Fig. S3a). However, a distinct fluorescence quenching is observed for the SP-treated QD films (Fig. 3b, Fig. S3b), where the bright fluorescent emission turns dark upon the treatment of SP. We reveal the non-radiative recombination loss of the QD films by proposing Förster energy transfer (FRET) between QDs and the dissociative (or unbonded) MC that exists in the SP molecules (Fig. 2a). In other words, the dissociative MC serves as an acceptor that induces the photoexcitation energy from the QDs (donor) to quench the QD emission. The dissociative MC in SP-treated QDs was demonstrated by the XPS measurements (Fig. S4), where the binding energies of Zn 2p peaks remain unchanged after thoroughly mixing SP with QDs. The dissociative MC does not bind to the QD surface, probably due to the reduced accessibility of MC to Zn^2+^ deriving from the large steric hindrance of oleic acid. Furthermore, we confirm the non-radiative energy transfer between QDs and the dissociative MC based on their optical characteristics. As shown in Fig. 3d, the dissociative MC exhibits a strong absorption peak centered at 583 nm, which has a broad overlapped area with the PL peak of QDs, thus favoring the FRET process. In other words, the excitation energy of the QDs (donor) can be facilely transferred to the dissociative MC (acceptor) upon photoexcitation (Fig. 3f), causing non-radiative recombination loss. In contrast, when the MC molecule binds onto the QD surface by the bonding interaction with Zn^2+^, there is a large blue shift of the absorption edge from ~700 to 575 nm, accompanied by a weakened absorption peak. This phenomenon was also observed previously due to the electron transfer from MC to the metal ions^48^. The resulting electron loss decreases the energy level of the highest occupied molecular orbital of MC, leading to an increased electron transition energy and a decreased electron transition quantity. Meanwhile, the stronger the electron-receptive capacity of the metal ion, the larger the blue shift of the absorption edge. For example, the lead ion has abundant empty orbits to receive the electrons, and the binding of MC to Pb^2+^ of the perovskite QDs largely shifts the absorption edge of MC to 524 nm, accompanied by the sharply weakened absorption peak intensity (Fig. S5). The blue shift of the MC absorption profile avoids the overlap with the QD PL spectrum (Fig. 3e). Therefore, the non-radiative FRET between QDs and the bonded MC is greatly suppressed (Fig. 3f), and the dark QD film recovers to a bright emission, as shown in Fig. 3c and Fig. S3c. Similar trends are also observed for the FAPbBr_3_ (FA: formamidine) perovskite QDs (Figs. S5 and S6) and InP/ZnS QDs (Fig. S7), validating the suppression of the FRET process to recover the QD fluorescence. As a comparison, the PbS QDs with an emission peak centered at 893 nm were adopted, which have no spectral overlap with the absorption spectrum of MC, thus the FRET process can be effectively suppressed (Fig. S8).

Furthermore, the time-dependent PL intensity variations of the SP-treated QD films under UV irradiation have been monitored in Fig. 3g and Video S1. Being consistent with the above results, the PL intensity of the QD films sharply decreases to ~44% of the initial value upon the treatment of SP due to the non-radiative FRET between QDs and the dissociative MC mixed in SP molecules (Fig. S9). When the SP-treated QD films are exposed to UV light for several seconds (e.g., 5 s), the PL intensity further declines to 8%, which can be derived from the increased amount of dissociative MC for more FRET loss (Fig. S9). However, the film PL intensity quickly increases to 31% by prolonging the UV exposure time to 1 min, indicating the bonded MC onto the QD surface for effective suppression of the FRET process. The PL intensity continuously increases to 95% with further extending the exposure time to 2 min and then remains unchanged by further elevating the time to 60 min, indicating the fast ligand exchange process from OA to MC within 2 min through the bonding interaction between MC and Zn^2+^ on the QD surface. To exclude the effect of UV irradiation on the QD PL intensity, the stability of CdSe/ZnS and FAPbBr_3_ QDs without SP additive was tested (Fig. S10), where no significant PL intensity decrease was observed for the QDs within 2 min. The result clarifies that the PL variations for QDs with SP arise solely from the photoisomerization. The PLQY variations during QD patterning were also recorded (Fig. 3h), in which the SP-treated QD films exhibit a remarkable decrease from 57% (pristine QD films) to 31%. Then, the PLQY value increases to 54% when the films are exposed to UV irradiation for 2 min. Subsequently, the PLQY value is further improved to 63% after the development process with octane. The continuous improvement in CdSe/ZnS QD PLQY after the developing stage can be attributed to the suppression of non-radiative FRET between QDs and the dissociative MC by removing the residual MC on the films with the developing agent. As a comparison, the PLQY of PbS QDs remains constant after the development stage due to no FRET process between PbS QDs and MC (Fig. S8). Notably, the PLQY value for developed QD patterns is higher than 110% of pristine QDs. The improvement in PLQY is much greater than previously developed QD patterns (Table S2). Generally, the PLQY for the developed QD patterns should recover to that of the pristine QD films by the suppression of the energy transfer loss. Thus, the higher PLQY than pristine QDs can be attributed to the additional passivation of QD surface defects through the bonding interaction between MC and the unsaturated Zn^2+^. As we know, there are inevitable dangling bonds on the QD surface, such as the unsaturated Zn^2+^ sites, which act as the QD surface defects and induce much non-radiative recombination. To further investigate the effect of the QD defects on the carrier recombination processes, the measurements of time-resolved PL (TRPL) were performed (Fig. 3i). The TRPL decay dynamics can be well-fitted by a bi-exponential function, where the fast time constant (τ_1_) relates to non-radiative recombination (e.g., trap-assisted non-radiative recombination, FRET non-radiative recombination) and the slow time (τ_2_) is associated with the radiative recombination^49,50^. The parameters of TRPL decay dynamics for each step were extracted and listed in Table S3. The obtained average lifetimes (*τ*_avg_) for the QD films are consistent with their PLQY variations. Specifically, the developed QD patterns exhibit a much longer lifetime (11.15 ns) than that of pristine films (9.81 ns), showing greatly improved radiative recombination. Moreover, we also demonstrated higher PLQY (91%) for developed FAPbBr_3_ perovskite QD patterns than the pristine QDs (84%) (Fig. S5), demonstrating good compatibility of our proposed strategy for highly emissive QD patterns with different types of QDs. The superior optical property of developed QDs paves the way to achieve high-quality QD pixels.

### Capacity for elaborated and ultrahigh-resolution QD pixels

**Fig. 4:**
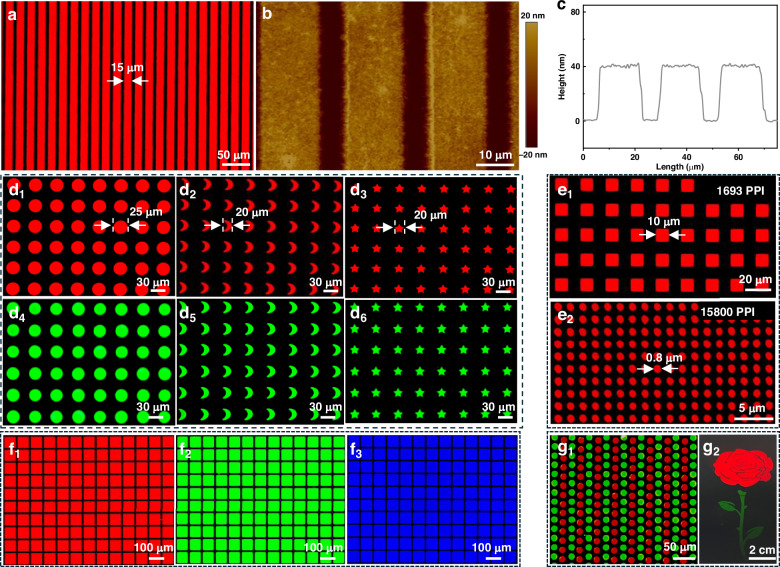
Optical and fluorescence images of QD patterns. **a** Fluorescence microscopy image of the QD strips and **b** the corresponding AFM image, and **c** the height profile of the QD strips extracted from AFM measurement. Fluorescence microscopic images of **d**_1_–**d**_6_ variously shaped CdSe/ZnS (red) and FAPbBr_3_ (green) QD pixels: **d**_1_, **d**_4_ circular, **d**_2_, **d**_5_ crescent, and **d**_3_, **d**_6_ pentagonal shapes, and high-resolution QD pixels with **e**_1_ 1693 and **e**_2_ 15,800 PPI. The multicolor QD pixels with **f**_1_ red, **f**_2_ green, and **f**_3_ blue emissions. **g**_1_ Exhibition of alternating QD pixels with red and green emission, and **g**_2_ their application in preparation of a vivid flower

The MC-capped QDs alter the solubility of QDs in nonpolar and/or low-polar solvents, thus matching the prerequisite for direct photopatterning. The utterly different solubility for pristine and MC-capped QDs is further verified in Fig. S11, where the pristine QDs quickly fall off from the substrate and rapidly dissolve in octane solvent. In contrast, the MC-capped QDs exhibit good robustness on the substrate without any discernible dissolution into the octane solvent, thus benefiting the preparation of multicolored, high-resolution, and high-fidelity QD patterns. Figure 4a shows the fluorescent image of patterned stripes by utilizing CdSe/ZnS QDs, which have a line width of 15 μm with an interval of ~5 μm. The height profile data extracted from the atomic force microscopy (AFM) image (Fig. 4b) reveal a consistent height of ∼40 nm for the stripes, indicating a good uniformity of the QD patterns. Meanwhile, we also fabricate QD stripes with a small line width of 1 μm (Fig. S12), showing a good capacity of our strategy to replicate the intricate patterns. In addition, we successfully fabricate variously shaped QD pixels of distinctive crescent and pentagonal shapes (Fig. 4d_1_–d_6_), which are rarely reported in previous studies due to their high complexities. Moreover, we demonstrate a very high fidelity (~100%) for these QD pixels with 25 μm (circular shape) and 20 μm (crescent and pentagonal shapes) in length, fully matching the size of the designed photomasks (Fig. S13). As reported, the perovskite (e.g., FAPbBr_3_) QDs are more sensitive to the patterning process than CdSe/ZnS QDs due to their fragile structures^36,37,51^. However, the complex crescent and pentagonal FAPbBr_3_ QD pixels are successfully achieved with a bright green emission (Fig. 4d_4_–d_6_), revealing the good applicability of our strategy to fabricate different types of QDs.

Besides the various pixel shapes, we also investigate the preparation of high-resolution QD pixels based on MC-capped QDs. As shown in Fig. 4e_1_, the square QD pixels with a size of 10 μm, corresponding to a resolution of 1693 PPI, are handily fabricated. To further verify the capacity for ultrahigh-resolution QD pixels, we designed an elaborate photomask to successfully achieve a minimal pixel diameter of ~0.8 μm (Figs. 4e_2_, S14), almost reaching the resolution limit of the designed masks for direct photopatterning. The fine QD pixels enable an ultrahigh resolution of 15,800 PPI, which represents the highest resolution achieved with a directly photopatterned method reported so far (Table S2). Meanwhile, we show the size-dependent QD patterning metrics (Figs. S14 and S15), where the QD pattern fidelity increases with the enlargement of the pattern size. In addition to the ultrahigh resolution, we showcase the successful fabrication of the multicolor patterns consisting of red, green, and blue QD pixels, corresponding to the three primary colors in displays (Fig. 4f_1_–f_3_). By successive patterning with red and green QD pixels (Fig. 4g_1_), we achieve a vivid picture of a flower with a large size of ~38 cm^2^ in the width and length of ~4.5 and 8.5 cm, respectively (Fig. 4g_2_). Moreover, we exhibit robust MC-capped QDs in the film with a high film retention ratio (Fig. S16), which enables the QDs' excellent resistance to the developing solution corrosion (Fig. S17). Accordingly, we demonstrate a good capability of our strategy to create orthogonal RGB pixel arrays (Fig. S18) for full-color displays. Besides the rigid substrates, we also demonstrate the good applicability of our method to prepare QD patterns on flexible substrates (e.g., polyimide and polycarbonate films) (Fig. S19). Consequently, we have demonstrated the excellent versatility of our strategy for the fabrication of small-sized, ultrahigh-resolution, high-fidelity, multicolor, and complex QD pixels. Meanwhile, the patterning method has good compatibility to fabricate elaborated QD pixels with conventional CdSe/ZnS and FAPbBr_3_ perovskite QDs, as well as wide applicability to rigid and flexible substrates.

### Performance of patterned electroluminescent devices

**Fig. 5:**
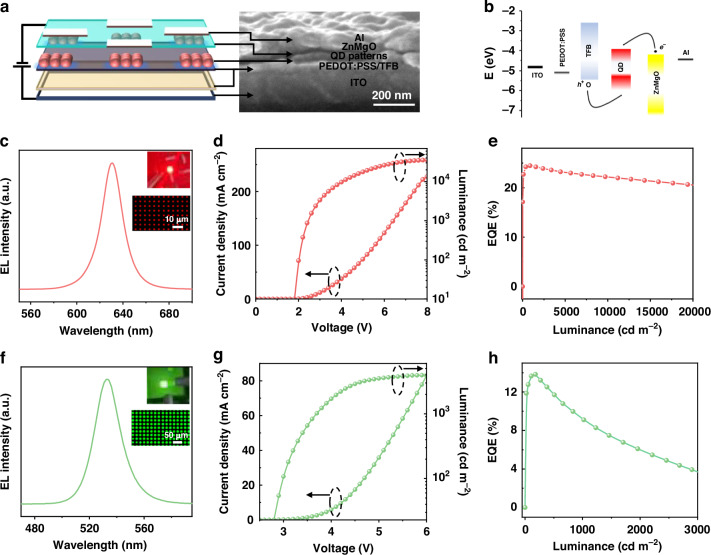
Device performance of patterned QLED. **a** Device structure and cross-sectional SEM image of the pixelated QLEDs and **b** the energy band diagram. **c** The EL spectrum for pixelated CdSe/ZnS QLEDs (inset: the operating picture of the devices and the corresponding EL image of the pixels). The pixelated CdSe/ZnS device performances: **d** current density–luminance–voltage (*J–L–V*) and **e** efficiency–luminance (*EQE*–*L*) curves. **f** The EL spectrum for pixelated FAPbBr_3_ QLEDs (inset: the operating picture of the devices and the corresponding EL image of the pixels) and their corresponding **g** *J*–*L*–*V* and **h** *EQE–L* curves

Inspired by the superior optical properties of the QD patterns, the QD light-emitting diodes (QLED) were further assembled to evaluate their electroluminescence (EL) performances. Benefitting from the good compatibility on different substrates, the QD pixels of the device were directly prepared on the hole transport layer of poly(9,9-dioctylfluorene-co-*N*-(4-butylphenyl)-diphenylamine) (TFB). The device structure is displayed in Fig. 5a, which consists of the functional layers of indium tin oxide (ITO), poly(3,4-ethylenedioxythiophene): polystyrene sulfonate (PEDOT: PSS)/TFB hole injection/transport layer, QD pattern emission layer, ZnMgO electron transport layer, and Al electrode, respectively. These layers can be observed from the device cross-sectional scanning electron microscopy, where the thicknesses of PEDOT: PSS/TFB, QD patterns, ZnMgO, and Al cathode are around 43, 40, 25, and 85 nm, respectively. As shown in Fig. 5b, when the devices are applied with a bias voltage, the electrons and holes will be driven to transport from the ZnMgO and TFB layers, respectively, and subsequently recombine radiatively in the QD patterns. The EL spectrum of CdSe/ZnS QD patterns with a high resolution of 6350 PPI (Fig. 5c and Fig. S20) shows an emission peak centered at 630 nm and a narrow full width at half maximum (FWHM) of 21 nm. The EL spectrum of CdSe/ZnS QD patterns is consistent with the profile of the OA-capped devices (Fig. S21), indicating that the QD optical property remains constant after capping with MC. Encouragingly, benefitting from the superior optical property of CdSe/ZnS QD patterns, as well as the isolation of ZnMgO and TFB layers with a perfluorodecyltriethoxysilane blocking layer, the patterned devices reveal a fantastic efficiency of 24.5% at a pixel resolution of 6350 PPI (Fig. 5d, e), representing the best-performing pixelated QLEDs among the direct photopatterning devices (Table S4). Meanwhile, the patterned devices exhibit a current efficiency of 28.7 cd A^−^^1^ and an operational lifetime (*T*_95_@1000 cd m^−^^2^) of 2214 h (Fig. S22). In addition, the MC-capped devices exhibit much higher device performances than the pristine ones (Fig. S23), which indicates the important role of the ligand exchange in improving the device performances, benefiting from the improved charge transport with a reduced QD-to-QD distance (Fig. S24). Besides, we also fabricated the pixelated FAPbBr_3_ QD devices with a typical structure of ITO/NiO_x_/TFB/FAPbBr_3_ patterns/1,3,5-benzinetriyl-tris(1-phenyl-1-H-benzimidazole) (TPBi)/LiF/Al. The perovskite devices exhibit an EL peak at 533 nm and a narrow FWHM of 22 nm (Fig. 5f). The achieved brightness and efficiency of the perovskite devices are 3961 cd m^−^^2^ and 13.8% at 1760 PPI, respectively (Fig. 5g, h), representing one of the best performances for pixelated perovskite devices (Table S5). Consequently, we have demonstrated high device performances for the fabricated QD pixels with a record EQE of 24.5% at 6350 PPI, being one of the highest efficiencies for the pixelated QLEDs. Meanwhile, we also reveal the wide applicability of the strategy for efficient pixelated perovskite devices, showing a great potential application of our strategy for efficient pixelated devices toward practical use.

## Discussion

In this work, we demonstrate a new principle of the photoisomeric transformation for high-quality QD pixels through the collaboration of SP and MC photoisomeric materials. We have revealed the mechanism of suppressed non-radiative energy transfer between QDs and the dissociative MC ligands for the enhanced emission efficiency of different types of QDs (e.g., red CdSe/ZnS, red InP/ZnS, green FAPbBr_3_, and near-infrared PbS QDs), and further verify this mechanism by exceptionally presenting the energy transfer-induced non-radiative loss for declined emission efficiency of the blue CdSe/ZnS QDs (Table S6, Fig. S25). We have demonstrated our approach for elaborate and ultrahigh-resolution QD pixels with high-resolution (15,800 PPI), high-fidelity (~100%), multicolor (RGB), and complex QD pixels. Moreover, we achieve high-performance electroluminescent pixels, which show a large luminance of 35,534 cd m^−^^2^ and a record efficiency of 24.5% at a pixel resolution of 6350 PPI, representing the most efficient ultrahigh-resolution QLEDs. Therefore, our strategy serves as an encouraging and effective approach for high-quality QD pixels and highly efficient pixelated devices, which contribute significantly to the QLED community. In addition to efficiency and pixel resolution, future research should also address the scalability and long-term stability of pixelated QLEDs, which are critical to promote their practical application.

## Materials and methods

### Materials

Lead bromide (PbBr_2_, 99.99%) and formamidine bromide (FABr, 99.99%) were purchased from Xi’an Polymer Light Technology Corp. The red and blue CdSe/ZnS QDs were purchased from Suzhou Xingshuo Nanotechnology Co., Ltd. Poly[(9,9-dioctylfluorenyl-2,7-diyl)-co-(4,40-(N-(4-s-butylphenyl)) diphenylamine)] (TFB) was purchased from American Dye Source. 1,3,5-benzinetriyl-tris(1-phenyl-1-H-benzimidazole) (TPBi) and LiF (99.99%) were purchased from Luminescence Technology Corp. N-N-Dimethylformamide (DMF, 99.8%), anhydrous dimethyl sulfoxide (DMSO, 99.9%), oleic acid (OA, tech. 90%), hexane (anhydrous, 95%), 1-butanol, and n-decylamine were purchased from J&K Chemical Co., LTD.

### Synthesis of FAPbBr_3_ QDs

FAPbBr_3_ QDs with an average size of ~10.1 nm (Fig. S26) were synthesized in air at room temperature. In detail, the perovskite precursor solutions were first prepared by dissolving 0.1 mmol of FABr and 0.2 mmol of PbBr_2_ in 0.5 mL of anhydrous DMF solvent. After that, 0.15 mL of precursor solution was dropped into a mixed solution, which consisted of 5 mL of toluene, 2 mL of 1-butanol, 0.3 mL of oleic acid, and 24.2 μL of n-decylamine. Then, the solution turned quickly from colorless to yellow-green. After stirring the solution for 10 min, the solution was centrifuged to obtain the precipitate. The precipitated FAPbBr_3_ QDs were purified by a sequential centrifuging process and finally dispersed in hexane.

### Preparation of the QD pixels

The QD pixels were prepared on glass and/or Si wafers, which were sequentially cleaned by de-ionized water, acetone, and isopropanol for 20 min each and then dried with nitrogen flow, followed by a treatment of UV-ozone for 25 min before use. The QD patterning mainly included three steps shown as follows: (i) QD film deposition: The mixed solution containing QDs (20 mg mL^−^^1^) and SP (2–8 *wt*% relative to QDs) was first stirred for several minutes and filtered through a 0.22 μm PTFE filter. Then, the mixture was spin-coated on a substrate at 2000 rpm for 40 s. (ii) UV exposure. The prepared QD films were exposed under UV lamps (365 nm, 15 mW cm^−^^2^, 2 min) with designed photomasks, consisting of different patterns. (iii) Developing process. The UV-irradiated QD films were further treated with a proper amount of low-polarity solvents, such as hexane and n-octane, to remove the unexposed QDs for patterns. For the preparation of red and green patterns and RGB patterns, the procedure is similar to the above steps, where the red, green, and blue QDs were patterned consecutively.

### Fabrication of pixelated CdSe/ZnS QD devices

The ITO glass substrates were successively cleaned with de-ionized water, acetone, and isopropanol and then dried with nitrogen flow, followed by a treatment of UV-ozone for 25 min. Then, PEDOT: PSS solution was spin-coated onto the substrates at 4000 rpm for 40 s and annealed at 140 °C for 15 min. After that, the substrates were transferred into a nitrogen-filled glovebox. The TFB solution (8 mg mL^−^^1^, in chlorobenzene) was spin-coated at 3000 rpm for 30 s, followed by annealing at 140 °C for 30 min. Then, the CdSe/ZnS QD solution (CdSe/ZnS QDs, ~12 nm, Fig. S27, 20 mg mL^−^^1^ in n-octane containing 2 *wt*% SP) was spin-coated at 2000 rpm for 40 s, followed by the pixel preparation process with the above steps. After the developing process, the perfluorodecyltriethoxysilane solution (0.5 mg mL^−^^1^, in n-hexane) was spin-coated on the patterned films and annealed at 100 °C for 10 min to isolate the TFB and ZnMgO transport layers. After that, ZnMgO solution (20 mg mL^−^^1^ in ethanol) was spin-coated at 2000 rpm for 30 s and annealed at 100 °C for 20 min. Then, the films were transferred to a high-vacuum thermal evaporator to deposit the Al cathode (85 nm) to complete the device fabrication process.

### Fabrication of pixelated perovskite QD devices

The ITO glass substrates were sequentially cleaned with de-ionized water, acetone, and isopropanol. After drying, the substrates were treated with UV-ozone for 25 min. Then, NiO_x_ solution (20 mg mL^−^^1^ in de-ionized water) was spin-coated onto the substrates at 4000 rpm for 60 s and baked at 120 °C for 10 min. After that, the substrates were transferred to a nitrogen-filled glovebox, followed by the TFB film preparation process as shown above. Then, perovskite QD solution (20 mg mL^−^^1^ in hexane containing 2 *wt*% SP) was spin-coated at 2500 rpm for 35 s, followed by the pixel preparation shown above. Then, the perfluorodecyltriethoxysilane solution (0.5 mg mL^−^^1^, in n-hexane) was spin-coated on the patterned films. After that, TPBi (45 nm), LiF (1 nm), and Al (100 nm) electrodes were successively thermally evaporated to complete the device fabrication process.

### QD pattern and device characterization

The XPS measurements were carried out using the equipment of Thermo Fisher Escalab 250Xi. FT-IR spectra were measured with Bruker Tensor 27. The AFM image of the QD strips was obtained using Bruker Dimension Icon 004. The UV–vis spectroscopy was conducted with a PerkinElmer spectrophotometer (Lambda 750S). The steady-state PL spectra and the PLQYs of the QD films were obtained using a spectrofluorometer of Edinburgh FS5. The Fluorescent images were captured by Olympus (STL-LEDRFA). The device performances were measured by an Ocean Optics system, which consists of a source meter (Keithley 2450), a spectrometer (QE Pro), and an integrating sphere (FOIS-1).

### DFT calculation

The DFT simulation was implemented in the Vienna ab initio simulation package (VASP). The projector augmented wave (PAW) method was used to treat the effective interaction of the core electrons and nucleus with the valence electrons, while exchange and correlation were described using the Perdew−Burke−Ernzerhof (PBE) functional. The cut-off energy is set at 400 eV for the plane-wave basis restriction in all calculations. K-points are sampled under the Monkhorst−Pack scheme for the Brillouin-zone integration (K-points were sampled using the Gamma Point). In all calculations, the forces acting on all atoms are <0.02 eV/Å in fully relaxed structures, and self-consistency accuracy of 10^−^^5 ^eV is reached for electronic loops.

## Supplementary information

Supplementary Information

Supplementary Video

## Data Availability

The data that support the findings of this study are available from the corresponding author upon reasonable request.
