# Supplementary material for: Highly efficient and ultrahigh-resolution quantum dot light-emitting diodes via photoisomeric transformation
Source: Light Sci Appl. 2026 Mar 9;15:157. doi: 10.1038/s41377-026-02246-0 (PMC12968083; doi:10.1038/s41377-026-02246-0)
Supplement: Supplementary file 1 — Supplementary Information [file 41377_2026_2246_MOESM1_ESM.docx]

**Supplementary Information for**

**Highly efficient and ultrahigh-resolution quantum dot light-emitting diodes via photoisomeric transformation**

Chenglong Wu^1^, Chengzhao Luo^1,^*, Yonghuan Huo^1^, Zixuan Chen^1^, Chengze Xu^1^, Xin Zhou^1^, Zhiyong Zheng^1^, Xinwen Wang^1^, Zhenwei Ren^1,^*, Yu Chen^1,2,^*

^1^School of Optoelectronic Science and Engineering & Collaborative Innovation Center of Suzhou Nano Science and Technology, Soochow University, Suzhou 215006, China

^2^National University of Singapore Suzhou Research Institute, Dushu Lake Science and Education Innovation District, Suzhou 215123, China

*Corresponding author. E-mail: czluo@suda.edu.cn; zhwren@suda.edu.cn; chenyu_ny@suda.edu.cn

Details of the ligand density calculations

The ^1^H-NMR spectra of pristine QDs (i.e., OA-capped QDs) exhibit a ratio of bound oleic acid to total oleic acid of around 91%. When the QDs were treated with MC, the ratio of bound oleic acid decreased from 91% to 71%, meanwhile, the ratio of free oleic acid increased from 9% to 29%, i.e., 20% of the OA ligands were removed from the QD surface. The ratio of MC to the total oleic acid is 21% for the MC-capped QDs, which is slightly larger than the reduced free oleic acid ratio of 20%. The TGA curve of pristine QDs shows a fast weight loss of ~19% from ~ 200 to 500 ^o^C due to the decomposition of the oleic acid. The number of QDs (treated as spherical shape) can be estimated from the equation: $N_{QD}=\frac{m_{QD}}{\frac{4}{3}\pi R^{3}\rho}$, where *m*_QD_ is the weight of the QDs, *R* is the radius of the QDs (~ 6 nm), and *ρ* is the QD density (~ 5.0 g cm^–3^). The surface area of the QDs is calculated by $S=4\pi R^{2}$. Then the OA ligand density can be calculated from the equation of $D=\frac{N_{OA}}{N_{QD} S}\times N_{A}$, where *D* is the ligand density, *N*_A_ is the Avogadro constant, and *N*_OA_ is the number of the OA ligand, which can be calculated from the OA weight ratio in the QDs. The calculation results are shown in Table S1, in which the total ligand number of MC-capped QDs is higher than that of pristine QDs, indicating that the MC ligand can effectively passivate the bare surface generated by OA removal.


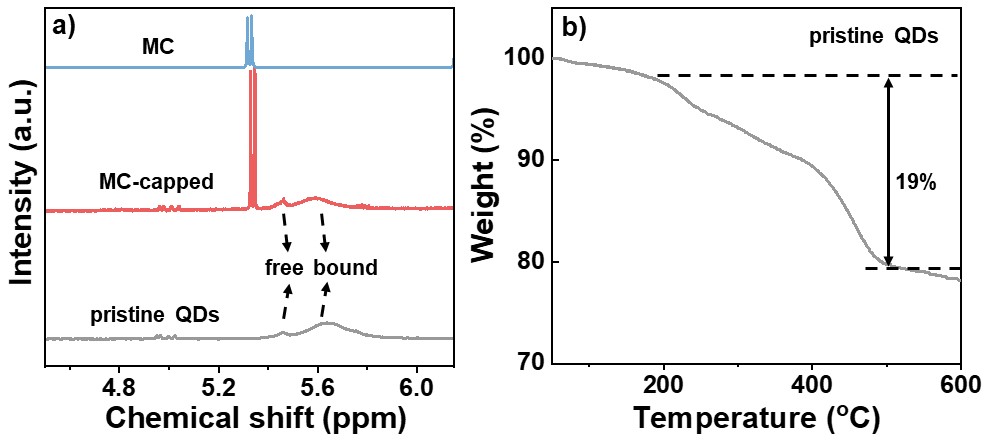


**Fig. S1** a) ^1^H-NMR spectra of pristine QDs with oleic acid (OA) ligand, MC-capped QDs, and MC molecule. b) Thermogravimetric analysis (TGA) of pristine QDs.


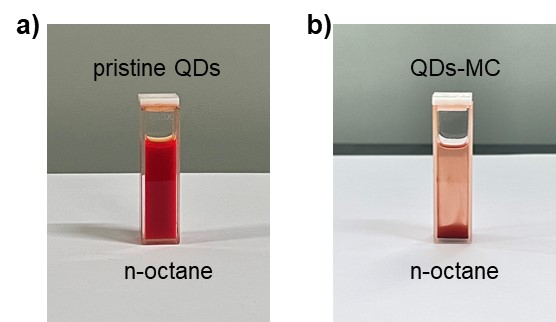


**Fig. S2** Photographs of a) pristine QDs and b) MC-capped QDs in n-octane solvent.


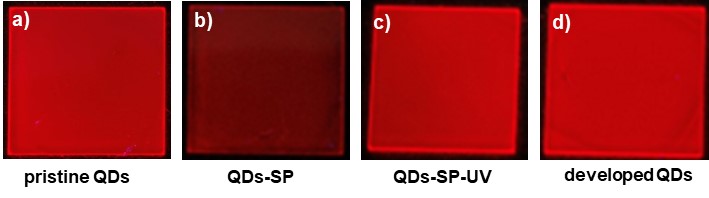


**Fig. S3** Fluorescence microscopic images of a) pristine CdSe/ZnS QDs, b) SP-treated CdSe/ZnS QDs, c) SP-treated CdSe/ZnS QDs after UV irradiation, and d) the developed CdSe/ZnS QD films.


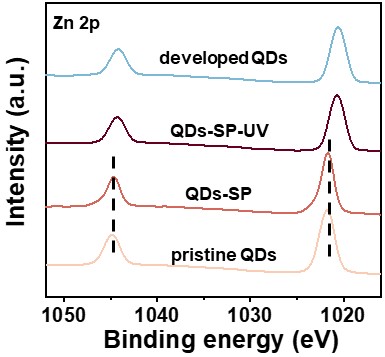


**Fig. S4** XPS measurements of Zn 2p signals in the samples of pristine QDs, SP-treated QDs, SP-treated QDs after UV irradiation, and the developed QD films.


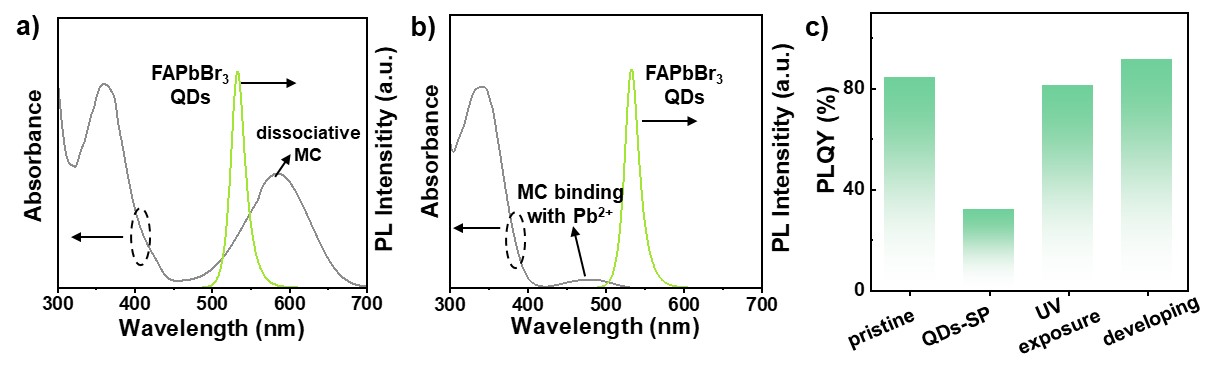


**Fig. S5** a) The overlapped spectra between the UV-vis absorption spectrum of dissociative MC molecules (UV irradiation: 2 s) and the PL spectrum of FAPbBr_3_ QDs, b) the nearly non-overlapped spectra between bonded MC molecules (UV irradiation: 2 min) and FAPbBr_3_ QDs, and c) the PLQY variations during the FAPbBr_3_ QD patterning process.


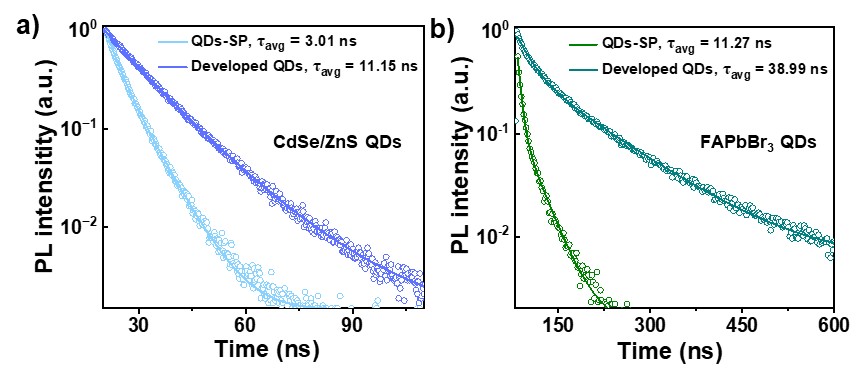


**Fig. S6** Time-resolved PL spectra of a) CdSe/ZnS and b) FAPbBr_3_ QDs with FRET process (SP-treated QDs, QDs-SP) and suppressed FRET process (developed QDs).The suppression FRET efficiency (*E*_s_) can be estimated from the equation of *E*_s_ = 1− (τ_sp_ /τ_dp_), where τ_sp_ and τ_dp_ represent the lifetime of the SP-treated QDs and developed QDs, respectively. The *E*_s_ values for CdSe/ZnS and FAPbBr_3_ QDs are 73% and 71%, respectively, where the slightly lower *E*_s_ for FAPbBr_3_ QDs may be induced by the very trace spectral overlap of the PL spectrum and the bonded MC absorption profile (shown in Fig. S5b).


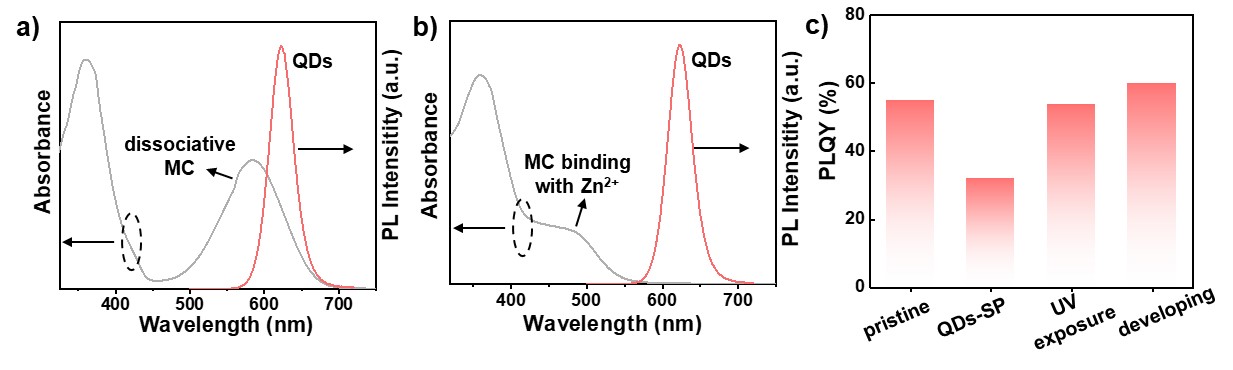


**Fig. S7** a) The overlapped spectra between the UV-vis absorption spectrum of dissociative MC molecules (UV irradiation: 2 s) and the PL spectrum of InP/ZnS QDs, b) the non-overlapped spectra between bonded MC molecules (UV irradiation: 2 min) and InP/ZnS QDs, and c) the PLQY variations during the InP/ZnS QD patterning process.


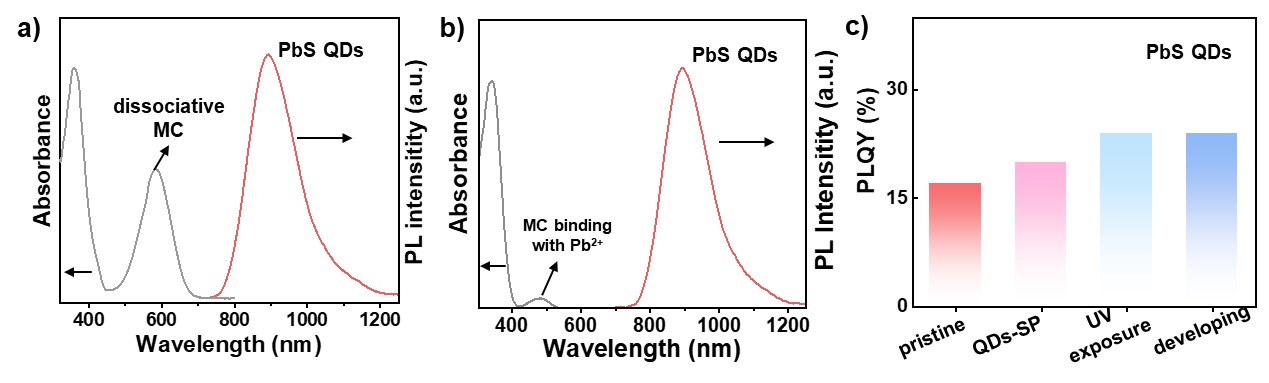


**Fig. S8** a) The UV-vis absorption spectra of dissociative MC molecules (UV irradiation: 2 s) and the PL spectrum of PbS QDs, b) the non-overlapped spectra between bonded MC molecules (UV irradiation: 2 min) and PbS QDs, and c) the PLQY variations during the PbS QD patterning process.


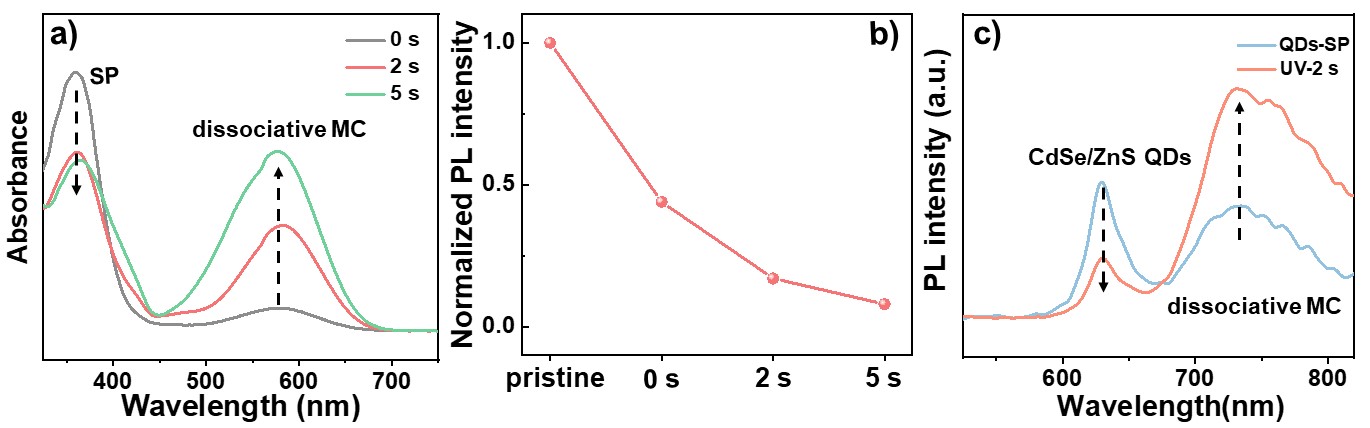


**Fig. S9** a) UV-vis absorption spectra of SP solution exposed to UV irradiation at different times, b) the PL intensity of pristine QDs and the PL intensity variations of SP-treated QD films under different UV exposure times, and c) the PL spectra of SP-treated QDs and the PL intensity variations under UV irradiation for 2 s.


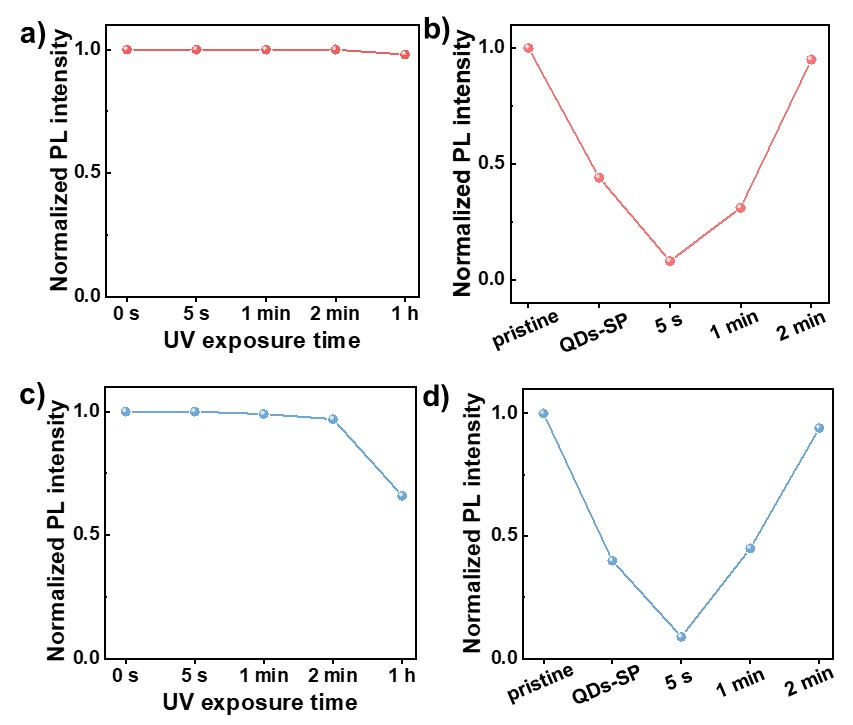


**Fig. S10** The PL intensity variations of a, b) CdSe/ZnS and c, d) FAPbBr_3_ QDs without a, c) and with b, d) SP additive under UV irradiation.


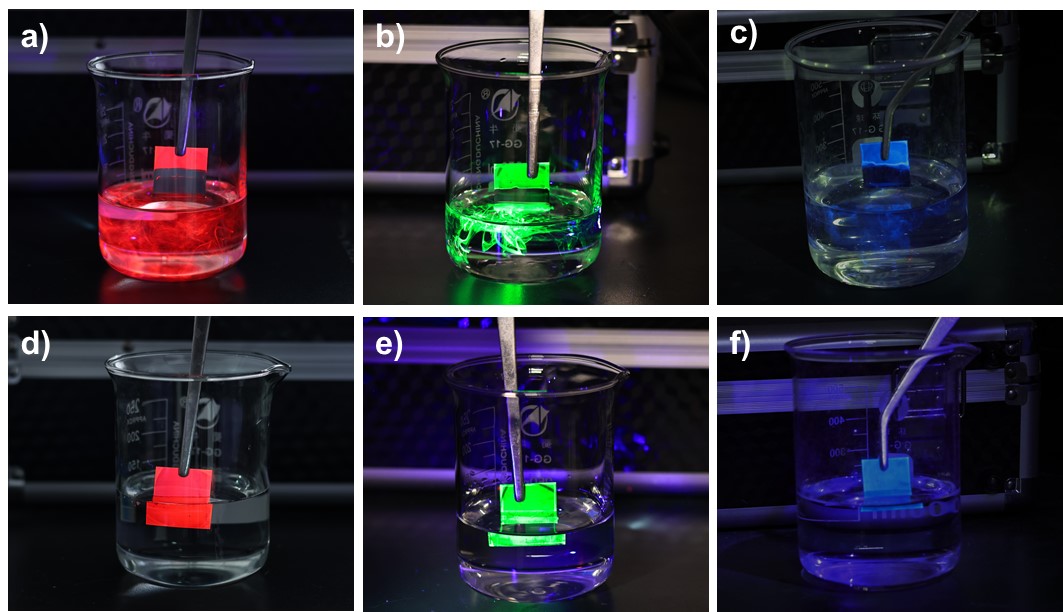


**Fig. S11** Fluorescence microscopic images of pristine OA-capped a) red CdSe/ZnS, b) FAPbBr_3_, and c) blue CdSe/ZnS QD films and MC-capped d) red CdSe/ZnS, e) FAPbBr_3_ and f) blue CdSe/ZnS QD QD films immersed in n-octane.


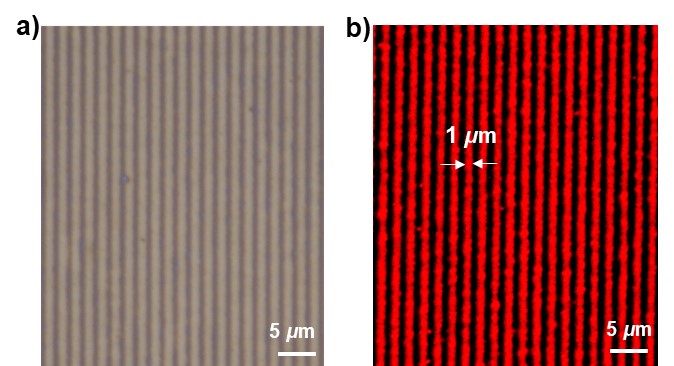


**Fig. S12** a) Optical and b) fluorescence microscopic images of CdSe/ZnS QD-patterned stripes with a very small line width of 1 *μ*m, reaching the resolution limit of the predesigned photomasks.


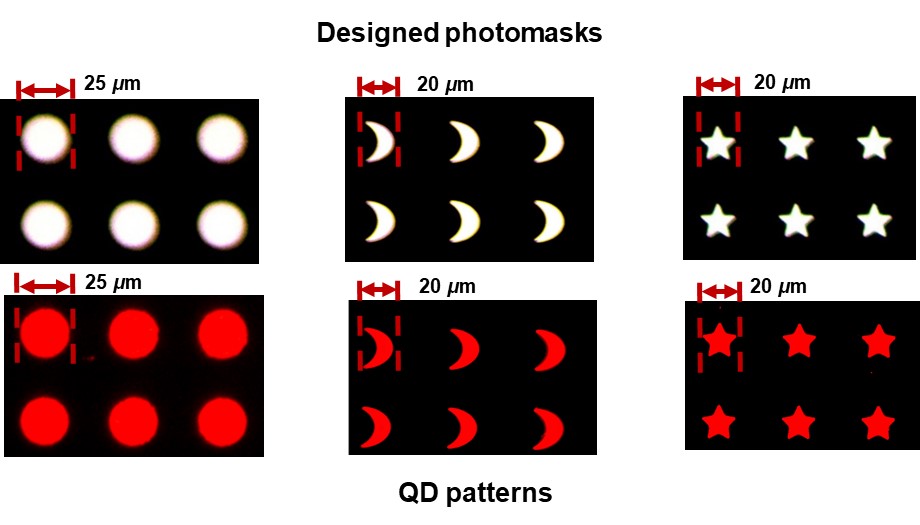


**Fig. S13** The size comparisons between the designed photomasks and the QD patterns. The perfect replication of the patterns from the designed masks reveals a high fidelity (~100%) of these QD patterns.


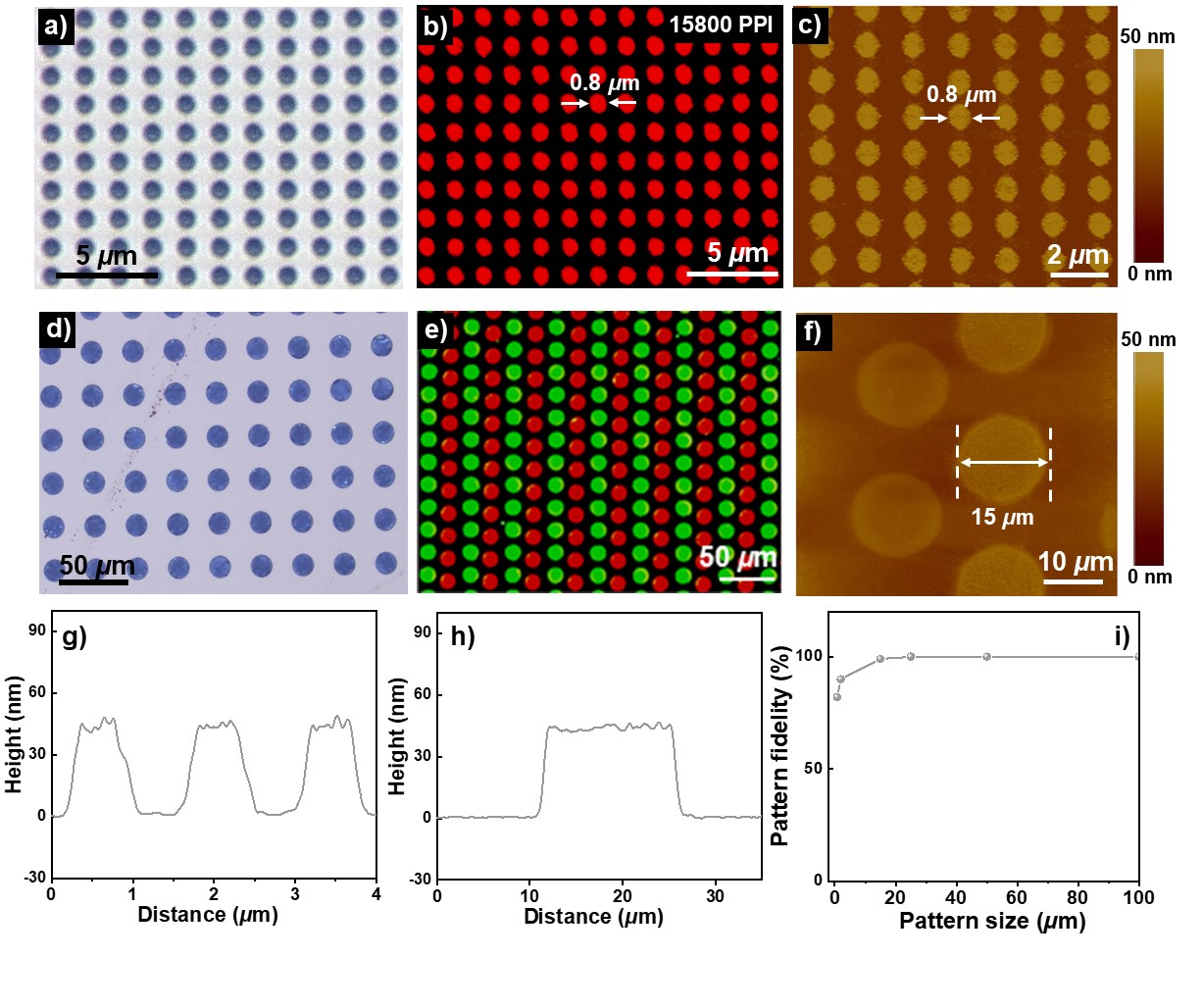


**Fig. S14** a, d) The optical microscopic images of the photomasks and b, e) the fluorescence microscopic, c, f) atomic force microscopy (AFM) images, and g, h) the height profiles of the high-resolution and multicolor patterns. i) The QD pattern fidelity at different pattern sizes. The submicron QD pattern (0.8 *μ*m) has a high fidelity of 82%, which increases to 90% at a size of 2 *μ*m, and further reaches ~100% with a QD pattern size of 15 *μ*m.


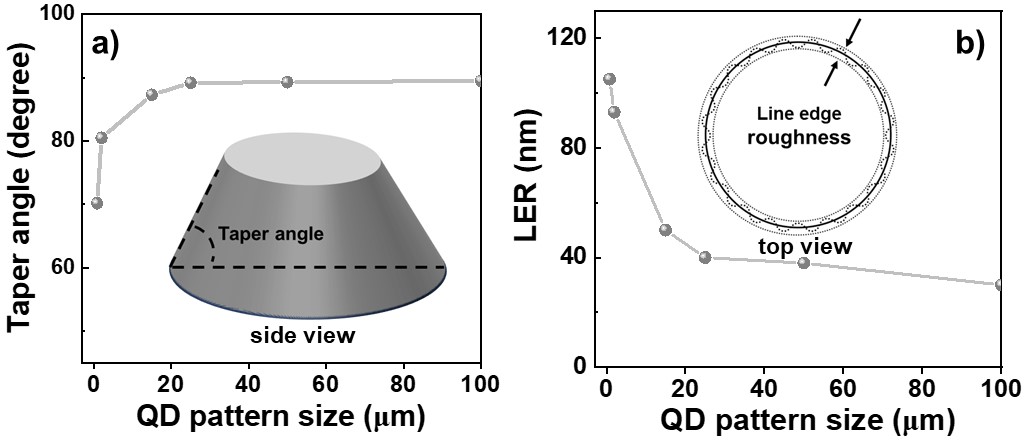


**Fig. S15** a) Taper angle and b) averaged line edge roughness (LER) of the QD patterns extracted from five samples that were prepared with 2 *wt*% of SP and a UV exposure dose of 1.8 J cm^−2^ (inset: the side and top views of the circular QD patterns).


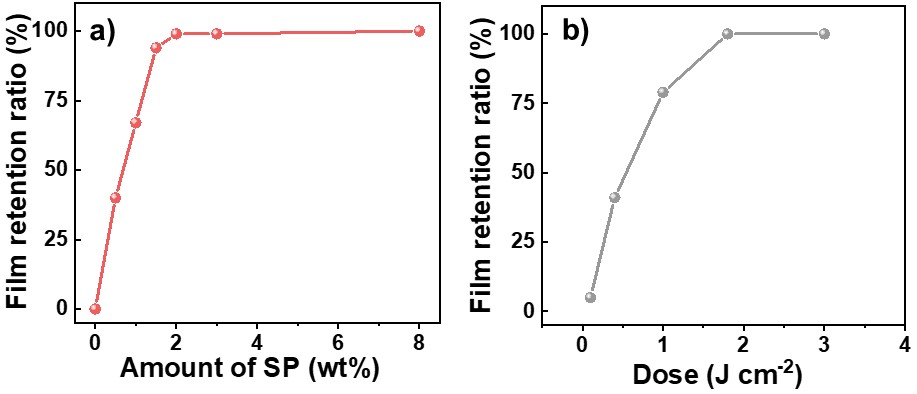


**Fig. S16** The film retention ratios versus a) SP concentration and b) UV exposure dose.


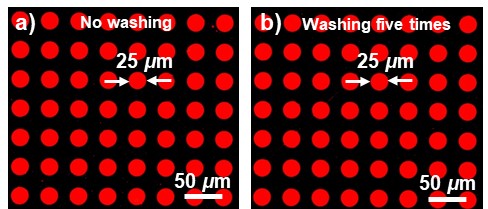


**Fig. S17** The fluorescence microscopy image of the MC-capped CdSe/ZnS QD pixels a) before and b) after washing with n-octane for five times.


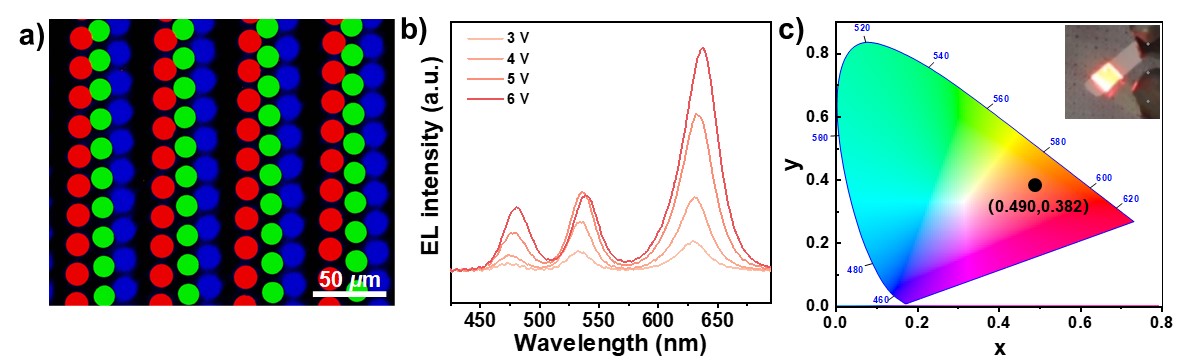


**Fig. S18** a) The fluorescence microscopy image of the RGB QD pixels, b) the EL spectra for pixelated RGB QLEDs, and c) the Commission Internationale de L’Eclairage (CIE) coordinate at 6 V (inset: the operating picture of the pixelated RGB devices).


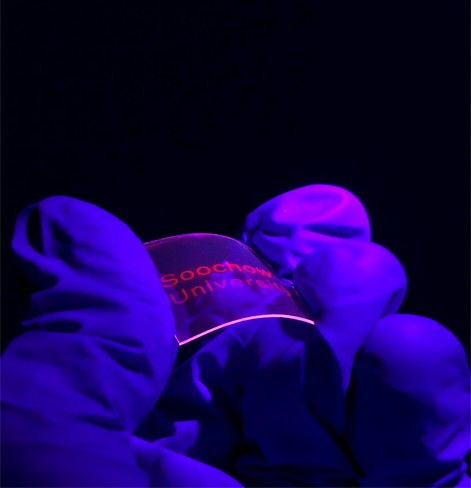


**Fig. S19** The red-emissive QD patterns prepared on a flexible substrate of polyimide.


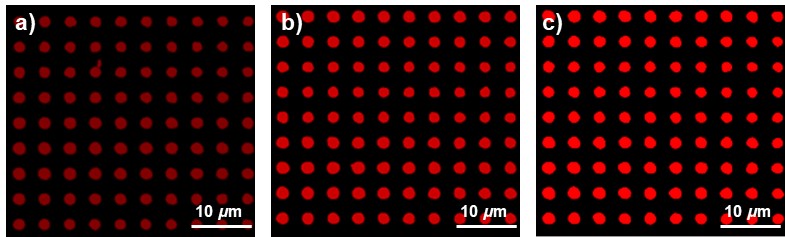


**Fig. S20** The EL images of the pixelated CdSe/ZnS devices under the applied voltages of 1.9, 2.0, and 2.1 V. The QD pixel image at higher voltages is too bright to be detected.


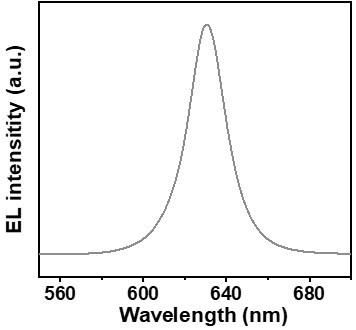


**Fig. S21** The EL spectrum for OA-capped pristine QDs-based devices.


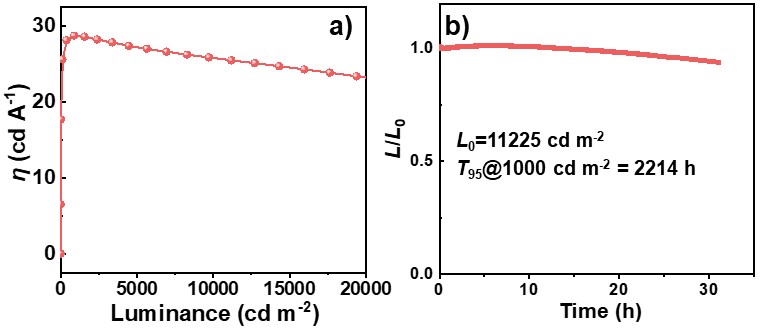


**Fig.** **S22** a) Current efficiency-luminance (*CE-L*) and e) operational lifetime curves of CdSe/ZnS QD patterned devices at a pixel resolution of 6350 PPI.


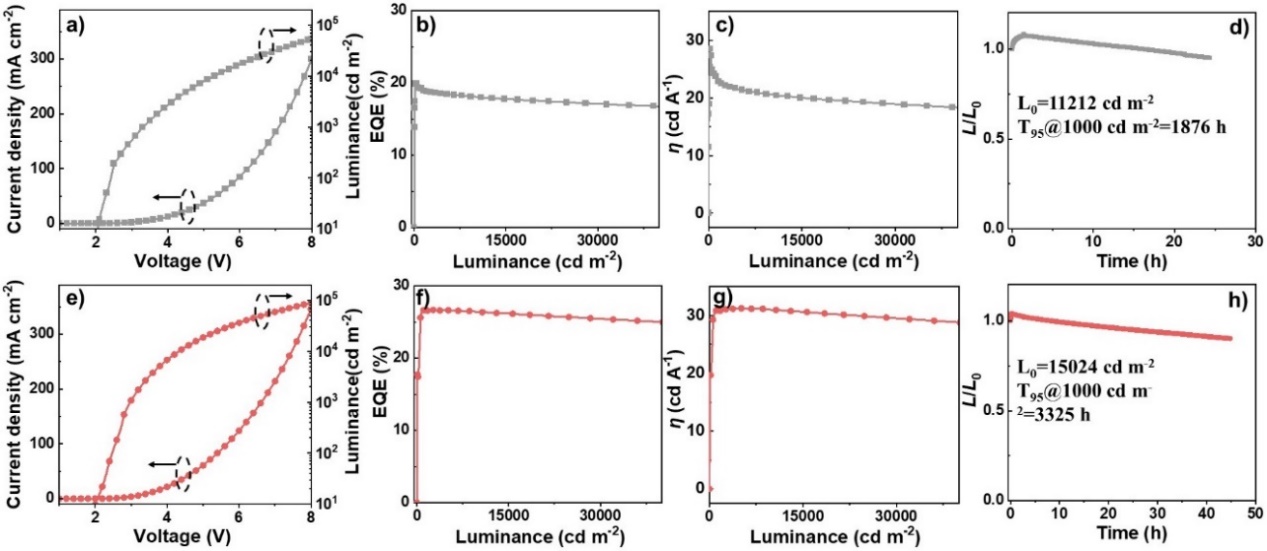


**Fig. S23** a, e) Current density-luminance-voltage (*J-L-V*), b, f) efficiency-luminance (*EQE−L*), c, g) current efficiency-luminance (*CE-L*), and d, h) operational lifetime characteristics for a-d) pristine and e-h) MC-treated devices.


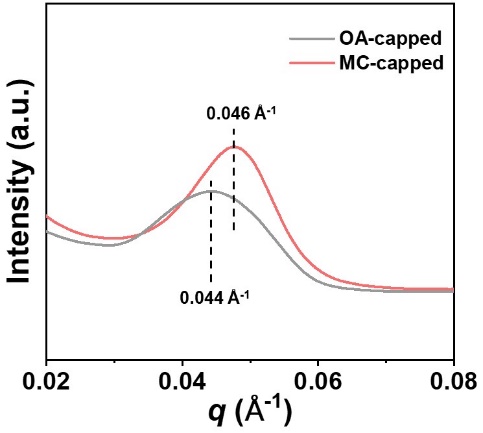


**Fig. S24** GISAXS measurement of the QD-to-QD distance of pristine QDs (OA-capped) before and after the ligand exchange with MC. The pristine QDs show a scattering peak located at 0.044 Å^−1^, corresponding to a QD-to-QD distance of 14.3 nm. Whereas the MC-treated QDs show a slightly larger scattering peak at 0.046 Å^−1^, indicating a shorter dot-to-dot distance of 13.6 nm, which favours the efficient charge transport after the ligand exchange.


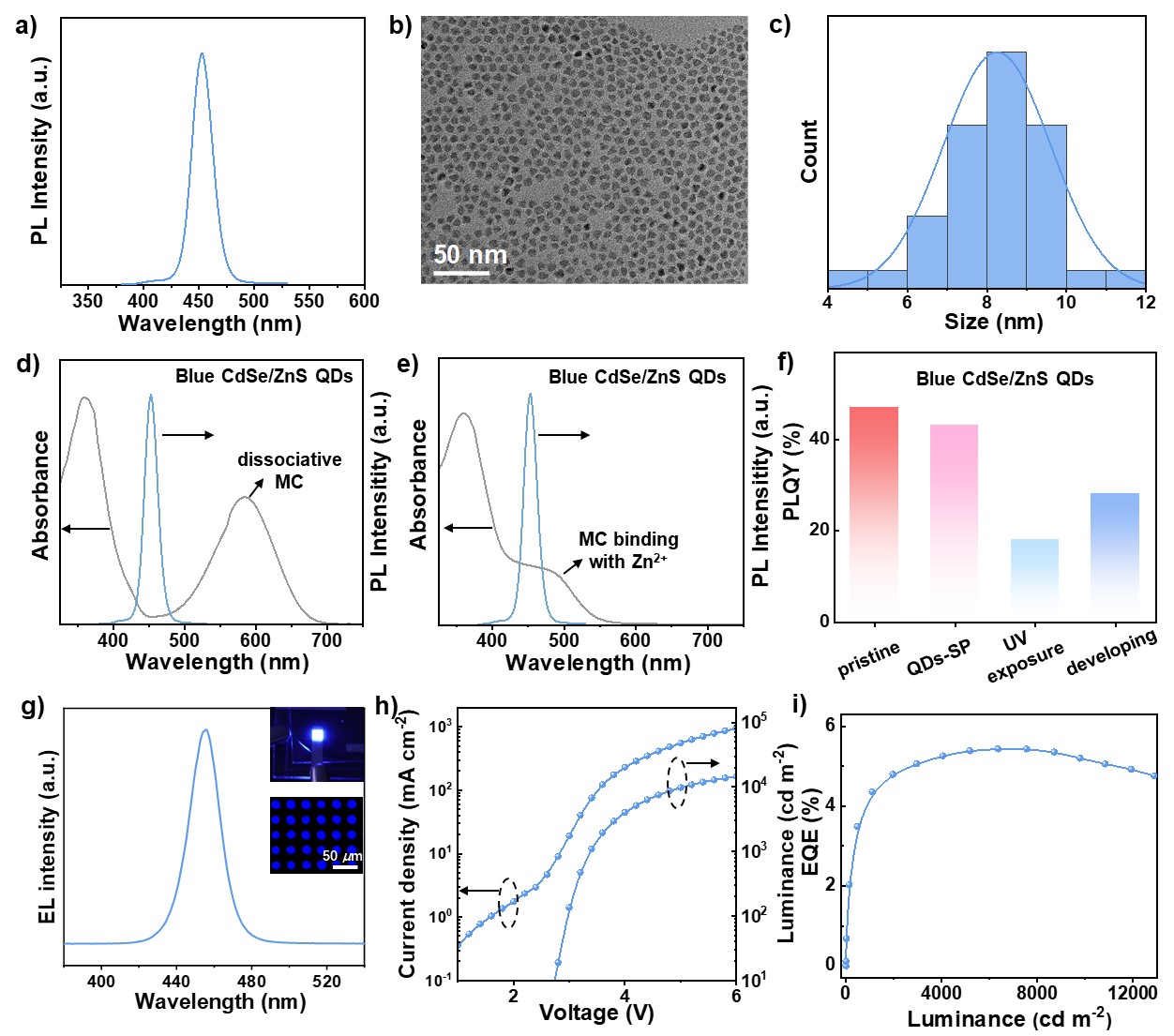


**Fig. S25** a) The PL spectrum, b) TEM image, and c) the size distribution of blue CdSe/ZnS QDs. d) The UV-vis absorption spectra of dissociative MC molecules (UV irradiation: 2 s) and the PL spectrum of blue CdSe/ZnS QDs, e) the overlapped spectra between bonded MC molecules (UV irradiation: 2 min) and blue CdSe/ZnS QDs, and c) the PLQY variations during the blue CdSe/ZnS QD patterning process. g) The EL spectrum for blue-emitting pixelated CdSe/ZnS QLEDs (inset: the operating picture of the devices and the corresponding EL image of the pixels) and the corresponding h) current density-luminance-voltage (*J-L-V*) and i) efficiency-luminance (*EQE−L*) curves.


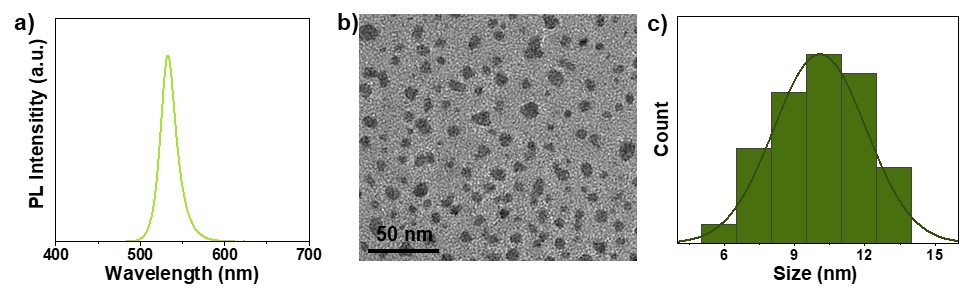


**Fig. S26** a) The PL spectrum, b) TEM image, and c) the size distribution of FAPbBr_3_ QDs.


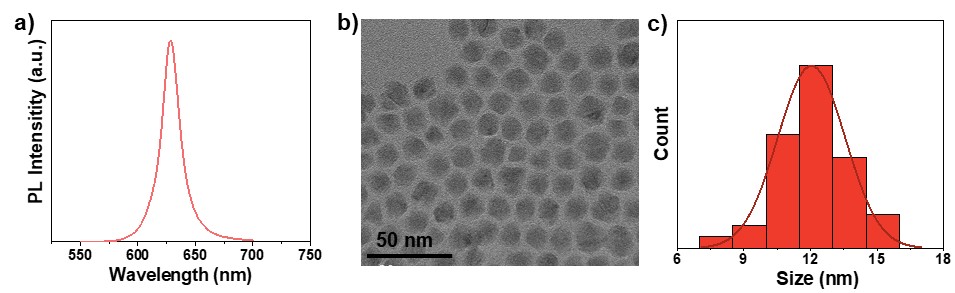


**Fig. S27** a) The PL spectrum, b) TEM image, and c) the size distribution of red CdSe/ZnS QDs.

**Table S1** Quantitative analysis of surface chemistry of pristine and MC-capped QDs.

|  | Pristine QDs | MC-capped QDs |
| --- | --- | --- |
| QD diameter (nm) | 12.0 | 12.0 |
| Surface area per QD (nm^2^) | 453.6 | 453.6 |
| Ligand density (OA, nm^–2^) | 4.0 | 3.2 |
| *N*_OA_ / *N*_QD_ | 1834.0 | 1467.2 |
| *N*_MC_ / *N*_QD_ | 0 | 385.1 |

**Table S2** Comparisons of the performances for CdSe/ZnS QD patterns and the pixelated QLEDs based on the direct photopatterning technique.

| Patterning mechanism | The PLQY ratio of developed QD patterns to pristine QDs | Maximum resolution (PPI) | EQE (%) and corresponding resolution | Published journals |
| --- | --- | --- | --- | --- |
| Ligand crosslinking | ~98% | ~1270 | 6.25 (50 PPI) | ACS Appl. Mater. Interfaces12, 42153 (2020) |
|  | ~95% | ~5080 | 14.6 (/) | Nat. Commun.  11, 2874 (2020) |
|  | ~90% | ~4200 | 12 (/) | Angew. Chem. Int. Ed. 61, e202202633 (2022) |
|  | ~92% | ~10000 | 16.3 (10000 PPI) | Nano Letters 24, 1254 (2024) |
|  | ~95% | ~12700 | 8.3 (/） | Adv. Mater. 34, e2205504 (2022) |
|  | ~102% | ~10580 | 20.05 (/) | Adv. Funct. Mater  2420829 (2025) |
|  | 90% | ~9540 | 22（/） | ACS nano.19, 14509–14520 (2025) |
|  | ~99% | ~6350 | 21.08% (6350 PPI) | Light Sci. Appl. 14, 251 (2025) |
| Ligand exchange | ~75% | ~8400 | 5.08 (/) | Adv. Mater. 32, 2003805 (2020) |
|  | ~97% | ~1000 | 4.9 (/) | Adv.  Mater. Interfaces 9, 2200835 (2022) |
|  | ~100% | ~1200 | 19.1 (1000 PPI) | Nano Lett. 23, 2000 (2023) |
|  | ~75% | ~508 | 0.4 (/) | ACS Energy Lett. 8, 4210 (2023) |
|  | 111% | 15800 | 24.5 (6350 PPI) | This work |

**Table S3** The parameters extracted from the fitting curves of TRPL decay dynamics for pristine, SP-treated, UV exposure, and developed CdSe/ZnS QDs films.

| Samples | *a*_1_ | *τ*_1_ (ns) | *a*_2_ | *τ*_2_ (ns) | *τ*_avg_ (ns) | χ^2^ |
| --- | --- | --- | --- | --- | --- | --- |
| Pristine QDs | 7.51 | 8.88 | 1.31 | 13.34 | 9.81 | 0.998 |
| QDs-SP | 663.75 | 2.85 | 12.32 | 6.65 | 3.01 | 0.997 |
| UV exposure | 10.69 | 6.46 | 3.42 | 12.01 | 8.54 | 0.999 |
| Developed QDs | 6.26 | 10.06 | 0.58 | 17.79 | 11.15 | 0.997 |

**Table S4** Comparisons of the pixelated QLED performances based on direct photopatterning technique.

| Patterning  technique | Types of QDs | EQE and corresponding resolution | Published journals |
| --- | --- | --- | --- |
| Photolithography | CdSe/ZnS | 8.2% (2116 PPI) | Adv. Mater.  35, 2303329 (2023) |
| Photolithography | CdSe/ZnS | 16.25%  (10000 PPI) | Nano Letters 24,  1254 (2024) |
| Photolithography | FAPbBr_3_ | 16% (907 PPI) | ACS Nano  18, 6896 (2024) |
| Photolithography | CdZnSe/ZnS | 9% (4000 PPI) | Adv. Funct. Mater. 35,  2420829 (2025) |
| Photolithography | FA_0.5_Cs_0.5_PbI_x_Br_3−x_ | 20% (3600 PPI) | Nature 640,  62–68 (2025) |
| Photolithography | InP | 22.6% (5080 PPI) | Nat. Commun 16,  4257 (2025) |
| Photolithography | CdSe | 21.08% (6350 PPI) | Light Sci. Appl. 14, 251 (2025) |
| Photolithography | CdSe/ZnS | 24.5% (6350 PPI) | This work |

**Table S5** Comparisons of the performances for perovskite QD patterns and the pixelated devices.

| Patterning mechanism | Perovskite QDs | The PLQY ratio of developed QD patterns to pristine QDs | Pixel size  (*μ*m) | Resolution (PPI) | EQE  (%) | Published journals |
| --- | --- | --- | --- | --- | --- | --- |
| Ligand crosslinking | FAPbBr_3_ | 63% | 60 | ~700 | 6.8 | Sci. Adv.  8, eabm8433 (2022) |
| Photolithography | CsPbBr_3_ | / | 5 | ~3031 | 3.9 | Adv. Mater.  35, 2303329 (2023) |
| Ligand crosslinking | CsPbBr_3_ | ~105% | / | / | ~2.5 | Sci. Adv.  9, eadi6950 (2023) |
| Ligand crosslinking | FAPbBr_3_ | 90% | 20 | ~907 | 16 | ACS Nano  18, 6896 (2024) |
| Ligand  exchange | FAPbBr_3_ | 108% | 10 | 1760 | 13.8 | This work |

**Table S6** The information of the employed QDs in this work and the emission efficiency before and after developing process.

| QDs | Emission peak (nm) | PLQY | |
| --- | --- | --- | --- |
|  |  | Pristine QD films | Developed QD patterns |
| Red CdSe/ZnS | 630 | 57% | 63% |
| FAPbBr_3_ | 533 | 84% | 91% |
| InP/ZnS | 622 | 55% | 60% |
| PbS | 893 | 17% | 24% |
| Blue CdSe/ZnS | 453 | 47% | 28% |

**Supplementary Video S1.** The time-dependent PL intensity variations of the SP-treated QD films under UV irradiation. The pristine and SP-treated QD films exhibit bright red emission under UV light at the beginning. Then a UV lamp was put on the SP-treated film and the lamp was turned on at the 26th s. After being irradiated for 5 s, the lamp was removed, leaving a darkened emission on the film. Then the film was continuously exposed to a UV lamp for 2 minutes from ~1 to 3 minutes, the darkened area recovered to a bright red emission.
